# Sugarcane mill mud-induced putative host (soybean (*Glycine max*))-rhizobia symbiosis in sandy loam soil

**DOI:** 10.1371/journal.pone.0293317

**Published:** 2023-11-02

**Authors:** Minori Uchimiya, Christopher M. DeRito, Anthony G. Hay

**Affiliations:** 1 Southern Regional Research Center, Agricultural Research Service, United States Department of Agriculture, New Orleans, Louisiana, United States of America; 2 Department of Microbiology, Cornell University, Ithaca, New York, United States of America; University of Florida, UNITED STATES

## Abstract

Domestic production of controlled-release, compost-based, and microbe-enhanced fertilizers is being expanded in the U.S. as a part of rural development. Sugarcane mill mud is a sterilized (≈90°C) agricultural byproduct in surplus that has received interests as a soil amendment in several Southern states, because of its high phosphorus and organic carbon contents. Addition of mill mud to sandy loam significantly increased the nodule formation compared to fertilized and unfertilized controls. Mill mud addition also resulted in pod yields similar to the fertilized control. Though not found in mill mud itself, mill mud additions correlated with an increase in soil Rhizobia as determined by deep 16S rRNA gene sequencing. We hypothesize that Firmicutes in sterilized mill mud induced Rhizobia that in turn enhanced soybean (*Glycine max*) growth. Collectively, mill mud enhanced the plant growth promoting bacteria when applied to a silt loam, although the relative influence of mill mud-derived bacteria, organic carbon, and nutrients is yet to be determined.

## Introduction

Historical investment is currently underway to expand the industrial-scale domestic fertilizer production in rural U.S. [1]. Those efforts are focusing on state-of-the-art technologies in biofertilizer production, including composting, anaerobic digestion, and microbial inoculation. Plant growth promoting rhizobacteria (PGPR) [2], especially for nitrogen fixation, have historically received interests for biofertilizer development. Various protocols have been proposed to inoculate seeds and soils with PGPR [3]. Controlled-release of inoculants has been a popular research topic for decades [4]. Although microbial inoculants are receiving renewed interests for fertilizer applications, one of the challenges that remains is viability throughout the product cycle from inoculation, storage, to efficacy in the agricultural field [5]. Given this major challenge, the use of diverse consortia has been proposed, as opposed to the traditional single-strain inoculants [6].

One promising organic amendment harboring potential PGPR is sugarcane mill mud. This byproduct represents all solid impurities in raw sugar production from extracted juice, and amounts to several million tons per harvest season [7]. Unlike the traditional use of bagasse byproduct to fuel the factory boiler [8], mill mud has not had a dedicated application. Although some factories apply mill mud to sugarcane fields [9], the large amount of legacy and newly generated mil mud streams is causing waste management problems at on-site waste pile/waste pond [7, 10]. In our previous report [10], mill mud byproducts collected from operating raw sugar production factories in Louisiana and Florida had high P (5.3 dry wt%) and C/N (25), and were dominated by Firmicutes. Firmicutes appeared to be the only bacteria that survived the high temperature (≈90°C) processing of sugarcane juice.

The following literature survey identified a missing linkage between the efficacy of mill mud and the role of its microbial community. Similarly to other organic amendments, mill mud can alter the master variables of soil chemistry (e.g., pH and dissolved organic carbon composition) [11] as well as soil compaction [12] and aggregate stability [13]. Those changes in soil physicochemical properties impact the microbial activity including the carbon use efficiency [14]. Soil microbiome is shaped by the environmental perturbations [15] and may control the bacterial and fungal composition of different sugarcane parts (leaf and stalk) [16]. Those complex interplays of soil chemistry and microbiology could result in enhanced sucrose yield and disease suppression [17] of sugarcane by mill mud [9] in cultivar-dependent manner [18]. In summary, no direct evidence is available to understand how mill mud may trigger crop growth enhancement. The present study aimed to understand the efficacy of sugarcane mill mud as a crop growth enhancer in weathered soil that needs amendments [11]. A particular focus was given to the quality enhancement (effects of mill mud on edible parts), rather than the overall (biomass) yield. A greenhouse experiment was designed to test how mill mud affects the soil microbial composition and soybean (*Glycine max*) growth, using chemical fertilizer as a control. Fresh mill mud and soil (without drying or other pretreatments) were used to understand the roles of native microbial communities on soybean yield and quality (nodule, seed pods). The active bacterial communities were characterized by deep sequencing of reverse transcribed 16S rRNA and complemented by shotgun metagenomics of total soil DNA to understand the functional potential of the soil microbial community.

## Materials and methods

### Greenhouse experiments

Top 15 cm of Langford silt loam soil (fine-loamy, mixed, active, mesic Typic Fragiudepts) was collected at Cornell agricultural field (42.47 longitude, -76.44 latitude). The field had been mowed but was left fallow for >10 years. Pots were filled in the field, and then transported to the Cornell greenhouse facility, where weights were normalized to 12 kg soil per 3 gallon pot. Soils were watered (≈3 L) twice a week for 2 weeks to acclimate to the greenhouse before mill mud addition. Prior to planting, soybean seeds (P09A62x; Pioneer brand Seeds, Corteva Agriscience, Johnston, IA) were incubated for 1 hr in a 10% slurry of soil separately collected from a nearby soybean field. The purpose of incubation was to inoculated seeds with native rhizobia [19] of soybean fields. The P09A62x variety is described to have high yield potential and white mold tolerance.

Fresh sugarcane mill mud was collected directly from the heated conveyor belt press filter of a commercial sugarcane processing factory in Louisiana during the 2021 harvest season. This sample is equivalent to “Mud-LA2” (from previous harvest season) in our previous report [10], where its chemical and microbial properties were described in detail. Air-drying, freezing, and other pretreatments could change the intact microbial community of biomass samples [20, 21]. Therefore, this greenhouse experiment was conducted using as-collected soil and mill mud samples without drying or sieving. Each treatment employed 10 replicates to account for plant, soil, and mill mud variabilities.

The treatments included a control (no fertilizer, watered once per week), fertigation (watered once per week with 300 ppm N; 21-5-20 (N-P_2_O_5_-K_2_O) all purpose LX; JR Peter’s, Allentown, PA) and 2% wet mill mud (calculated on a g/g dry soil and mud basis; watered once per week) treatments. Organic residues are commonly land applied in the range of 1–5% depending on a variety of factors that include plant needs, soil fertility, and the nutrient composition of the amendment [22]. All treatments (control, fertigation, and 2% mill mud) received supplemental high intensity discharge lights for 14 h per day.

For mill mud treatment, as-received mill mud was mixed into the soil by hand and brought up to field capacity (17 wt%) with tap water. After three days of acclamation, each pot was planted with 9 soybean seeds in a 3×3 grid approximately 5 cm apart. Germination was monitored every 2–3 days for two weeks; then, plants were thinned to three plants per pot and watered as needed. Once plants entered the full bloom stage (~60 days after planting), pod production was measured every 10 days until most plants lost ~75% of their leaves. Plants were harvested after 115 days. Intact plants were gently removed from moistened soil to preserve root structure and attached nodules. Shoots were then cut at soil level to allow for measurement of above-ground biomass.

Residual soil attached to roots was gently removed using distilled water in a squirt bottle and nodules were separated and counted. All materials were autoclaved after the greenhouse experiment.

### RNA extraction for marker gene (16S rRNA) sequencing

At the end of greenhouse experiment, 3 replicate soil samples were taken per pot, compounded and stored at -80°C until extraction. RNA was extracted from ≈200 mg of the compounded root soil using a Quick-RNA fecal/soil microbe microprep kit (Zymo, Irvine, CA). Extracted RNA was quantified using a Qubit HS RNA assay kit (Invitrogen, Thermo Fisher Scientific, Waltham, MA), and reverse transcribed using an iScript cDNA synthesis kit (BioRad, Hercules, CA). An aliquot of the resultant cDNA (1 ng) was used as template for PCR amplification targeting the V4 region of the 16S rRNA gene [23, 24] using barcoded primers (515F 5’-CCTACGGGAGGCAGCAG-3‘ and 806R 5’-GGACTACHVGGGTWTCTAAT-3‘) and high-fidelity polymerase (New England BioLabs, Ipswich, MA). The PCR products were purified and normalized on a Sequalprep plate (Applied Biosystems, Waltham, MA), and then pooled. Ten pg of phiX was added as a control. Paired-end sequencing (2 × 250 bp) was carried out on an Illumina MiSeq sequencer at the Cornell Sequencing Center (Ithaca, NY).

### DNA extraction for metagenome

DNA was extracted from ≈200 mg of the compounded root soil using an ultra-clean microbial DNA isolation kit (MO BIO, QIAGEN, Germantown, MD), and extracts were purified using a OneStep PCR inhibitor removal kit (Zymo). DNA was quantified using a Qubit dsDNA assay kit (Invitrogen). Metagenome libraries were prepared using an Illumina DNA prep kit (Illumina, San Diego, CA). Prior to sequencing (Cornell Sequencing Center), library inputs (metagenome, 16S rRNA) were purified and normalized using a SequalPrep normalization plate kit (Applied Biosystems, Waltham, MA).

### Sequence processing

Raw sequences were uploaded to QIITA and processed through the standard workflow [25]. Briefly, they were trimmed, chloroplasts and mitochondrial sequences were filtered as were all singletons. Sequences that were 97% similar or more were grouped as a single operational taxonomic unit (OTU). The resultant OTU Deblur table was used as the basis for alpha and beta diversity analysis. Shotgun metagenomic sequences were uploaded to MG-RAST and processed through the standard workflow [26].

### Statistical analyses

Statistical comparisons were performed on three randomly chosen samples per treatment that yielded both usable 16S and shotgun metagenome libraries since not all libraries for all samples yielded usable sequences. Both the PCA and the heatmap were generated in Clust-Vis [27] via implementation of the pcaMethods and pheatmap packages in R using default settings. The PCAs were calculated using single value decomposition with imputation. The ellipses on the PCAs delineate the 95% confidence intervals in which a new observation from the same group would likely be found. The heatmap rows were scaled to unit variance (within row values were divided by standard deviation so that each row has variance equal to one). Cladograms above each heat map are graphical representations of quantitative similarity between samples as calculated using complete linkage clustering: the shorter the vertical line between samples on the same node (sharing a horizontal bar), the more similar they are. Between group/clade statistical comparisons (p<0.05) of individual measures were determined via analysis of variance (ANOVA) followed by Tukey’s honestly significant difference (HSD) using iCalcu [28]. False discover corrections were made using the Bonferroni method when testing multiple hypotheses [29].

## Results and discussion

### Nodule and seed pod growth enhancements by the organic amendment

**Fig 1:**
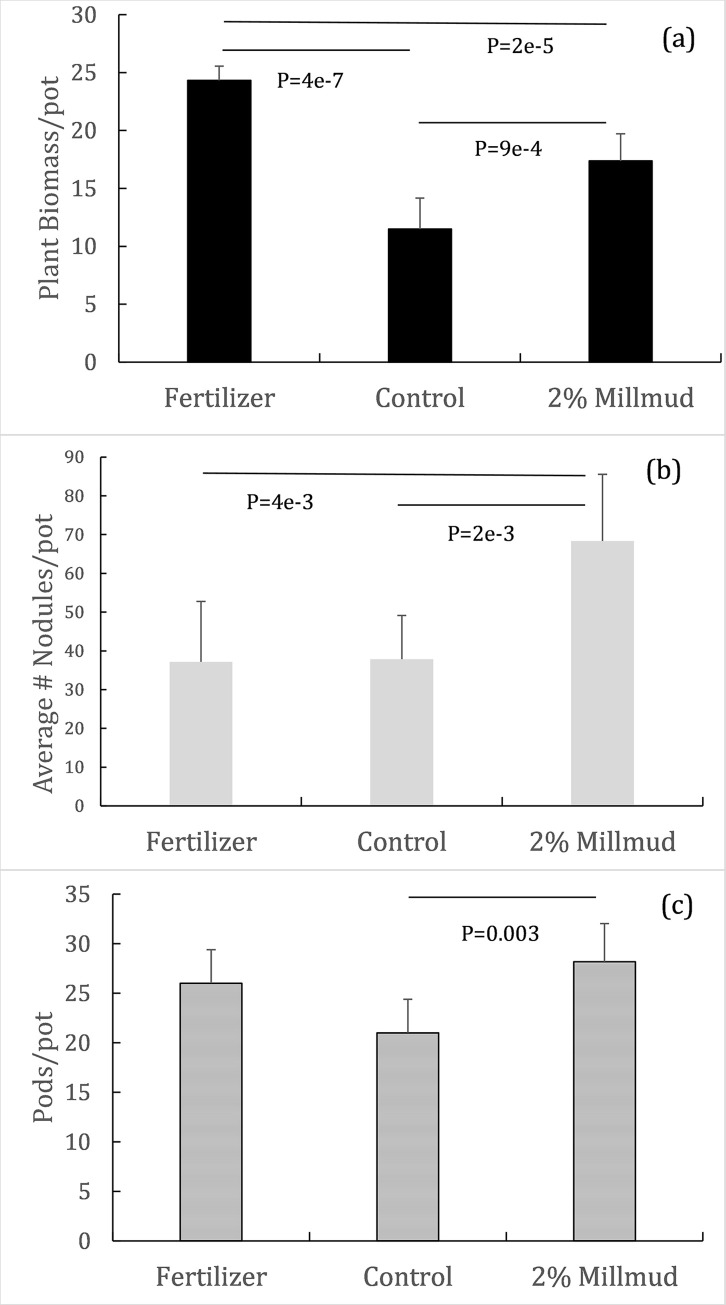
Soybean biomass (a) and root nodule (b) yields and seed pod counts (c) with (far left bars) and without (middle bars) chemical fertilizer, and with 2 wt% mill mud (no additional fertilizer, far right bars). Error bars represent one standard deviation.

Fig 1 presents effects of each treatment (mill mud, fertilizer, and control) on biomass, nodule, and seed pod yields from soybean pot experiments. Sugarcane mill mud significantly (p<0.05 denoted by horizontal lines in each panel) enhanced nodule growth, compared to the control and fertilizer treatment (Fig 1B). In addition, mill mud increased seed pod count, relative to the control (Fig 1C). Above ground biomass yield (shoots including pods) was significantly different for each treatment, increasing from control, mill mud, to fertigation (Fig 1A). Those results indicate mill mud’s ability to enhance the bean yield, rather than the biomass. A large number of agronomy experiments in the literature focused on the overall (aboveground biomass) yield. However, the yield of edible part (bean) provides a measure for the amendment’s effects on the quality of the soybean and other food crops. Because nitrogen fixation is a frequent target for biostimulants [3] and other approaches to microbially enhance crop quality/yield, subsequent sections will focus on root community structures and functions.

### Phylogenetic characterization of samples: Phylum

**Fig 2:**
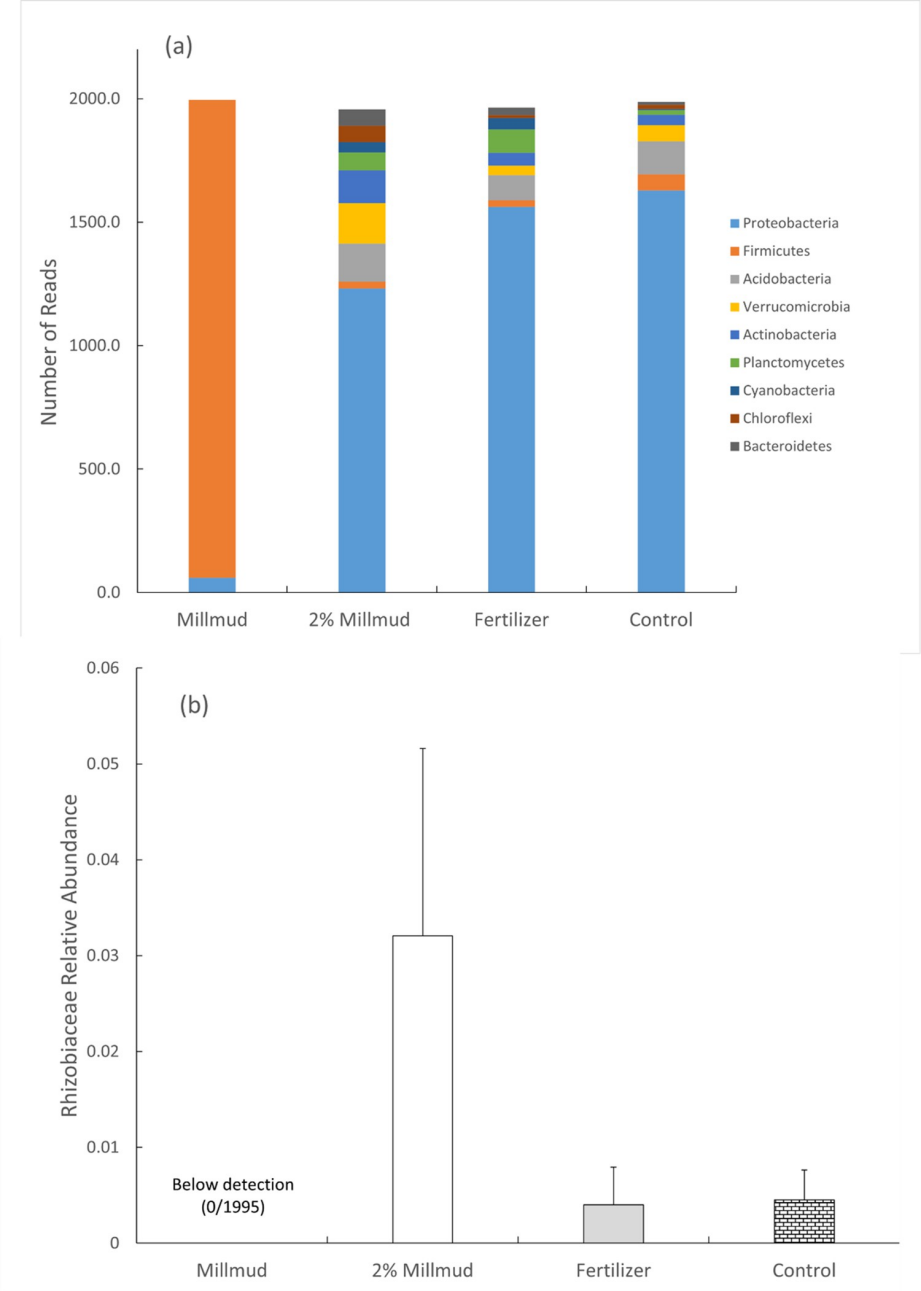
Phylum level affiliation of reads binned at the OUT level (a). Differences in relative abundance of Rhizobiaceae (b). Error bars represent one standard deviation.

Six phyla accounted for ≈95% of all sequence reads (Proteobacteria (58), Firmicutes (22), Acidobacteria (5), Verrucomicrobia (4), Actinobacteria (3), and Planctomyces (2)). In total, nine phyla with relative abundance greater than 1% accounted for 99% of all reads including Cyanobacteria (1.3), Chloroflexi (1.3), and Bacteroides (1.2) (Fig 2A). The clearest difference between samples was for the fresh mill mud (as-received, before soil amendment), which had 97% firmicutes reads, as opposed to the soils, which had 1–3% firmicutes reads regardless of treatment. Although raw mill mud is included in the later analyses of alpha and beta diversity for comparisons, the majority of subsequent sections is focused on the contrasting results for the different soil treatments.

### Order

15 orders were represented in soils with reads greater than 1%, accounting for 72.6% of all reads. Of these, 4 orders had significantly different average relative abundances between the 2% mill mud treatment and the fertilized soil/controls when corrected for false discovery (p<0.003). Myxococcales, Rhizobiales, and Chthoniobacterales were 2–4 times higher in the 2% mill mud amended soil, whereas Pseudomonadales were 8–12 times lower in mill mud amended soils.

### Family

At the family level, 19 taxa present at 1% or greater accounted for 64.4% of all reads. Of these, Leuconostocaceae and Lactobacilliales were only detected in raw mill mud, and were removed from analyses of soil bacteria. Of the remaining families in the top 20, only Pseudomonadaceae was significantly lower in the 2% mill mud soil than the control (by 8.7-fold) or fertilized soil (12.6-fold) when correct for false discovery (p<0.0025). Chthoniobacteraceae, an uncultured member of the Verrucomicrobia phylum, on the other hand, was 2–3 times more abundant in 2% mill mud soil than either of the other soils (p<0.0025), as was an uncharacterized member of the order Myxococcales, although the difference was only significant when compared to the control soil. Little is known of the roles of either family in the soil environment [30], although Mixococcales reportedly increase in soils receiving organic amendments [31].

The increase in nodules for plants in mill mud amended soils led us to hypothesize that the Rhizobiales family might be present at greater levels in 2% mill mud amended soil since some members of this family form symbioses in the nodules of soybeans [2, 32]. The relative abundance of Rhizobiales was indeed higher in mill mud amended soil (~8x) compared to the control (p = 0.037) and fertilizer (p = 0.036). The p value in this case was not corrected for false discovery rate since it was a single hypothesis based on the observed nodules. Rhizobiaceae OTUs were not detected (0 in 1995 reads) in mill mud itself (Fig 2B).

### Genus

At the genus level, 15 taxa that had relative abundances of 1% or more accounted for 49% of all reads. Of these, *Lactobacillus* and *Leuconostoc* were only detected in raw mill mud, and were removed from analyses of soil bacteria. The relative abundance of three genera in the top 15 were significantly different. Relative to the 2% mill mud treatment, Pseudomonas was higher in the fertilized soil (8.5-fold) and the control (12.6-fold, p<0.0033). One novel Mycoccales genus in the top 15 genera was 2–3 times higher in mill mud amended soils, but the difference was only significant when compared to the unfertilized control. Although *Rhizobium* was not in top 15, it was found to be 3–4 times more abundant in mill mud amended soil relative to the control (p = 0.001), and fertilizer treatment (p = 0.002). Also of note was the presence of 2 novel Rhizobiales genera that were more abundant in 2% mill mud amended soil. Although the differences were not significant after FDR correction, the trend suggests that related genera appear to respond to mill mud in a similar fashion.

### Alpha and beta diversities of microbial 16S rRNA gene community

**Fig 3:**
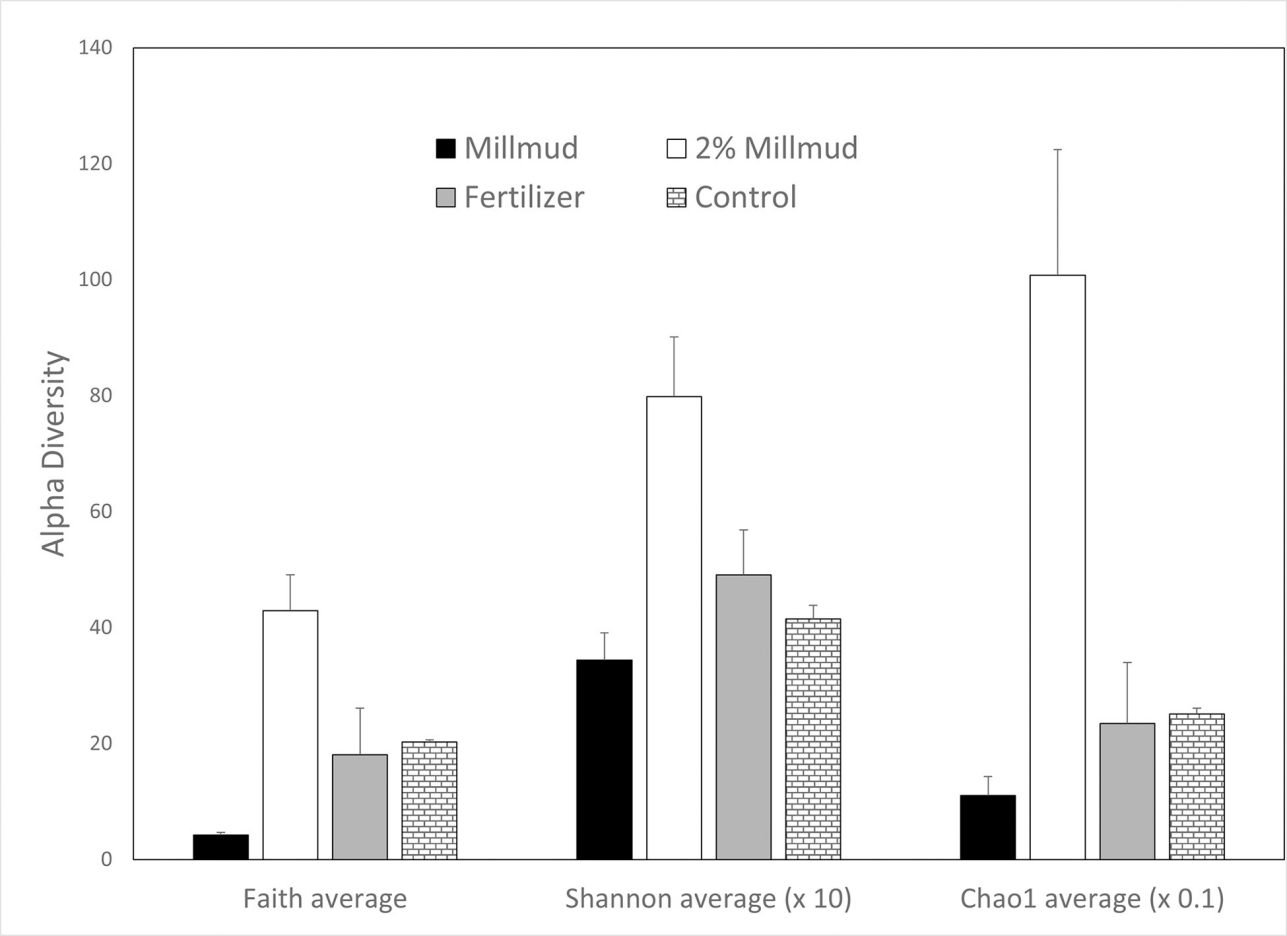
Alpha diversity OTUs (97% similarity) of 16S rRNA transcripts for mill mud alone, and 3 soil treatments: 2 wt% mill mud, chemical fertilizer, and control. Error bars represent one standard deviation. The 2% mill mud amendment was significantly different from all other treatments for each index (p<0.05).

Fig 3 compares OTU level alpha diversity of the 3 treatments employed in the pot experiments: 2% mill mud, fertilizer, and control. Mill mud (before soil application; black bars in Fig 3) is also presented for comparison. Phylogenetic diversity (Faith), evenness and richness (Shannon), and evenness/richness with emphasis on rare taxa (Chao1) were consistently the highest in mill mud-amended soil (2% mill mud, clear bar in Fig 3). Although there is some debate over the metrics of diversity [33], ecosystems with higher alpha diversity are generally considered to be more stable and resilient [34, 35].

**Fig 4:**
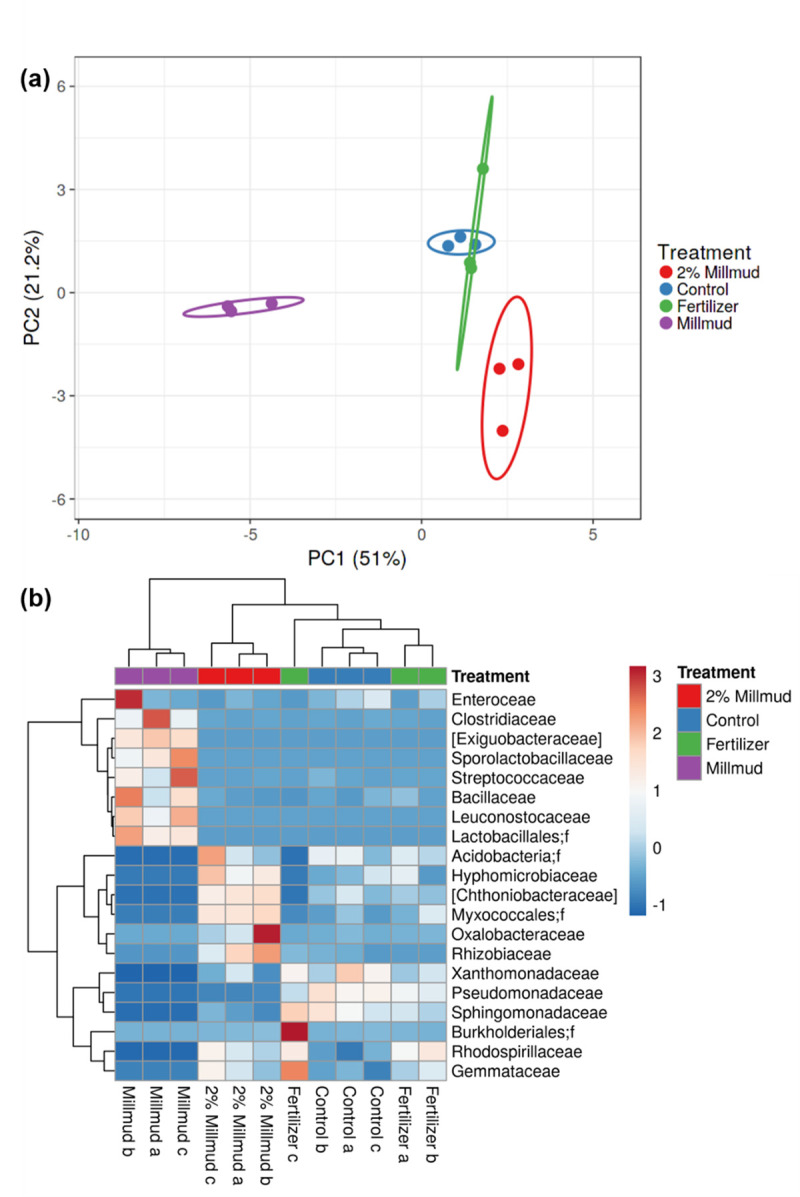
Principal components (a) and linkage clustering heatmap (b) from beta diversity analysis of 23 most abundant orders greater than 1% relative abundance of 16S reads. In both panels, soil treatments are color-coded for 2% mill mud (red), control (blue), and liquid fertilizer (green); fresh mill mud (purple) is shown as the control for the amendment before soil application. In (b), families that have no cultured representatives ([]) and uncharacterized families (;f) are denoted in row labels.

In addition to the inherent diversity within communities (alpha), the differences in diversity between communities (beta) can be a useful tool for understanding the potential impacts of different treatments [33]. Fig 4 presents a principal component analysis based on the relative abundance of the 23 orders that were present at >1% and accounted for 72% of all sequenced reads. In Fig 4A, mill mud-amended soil (2 wt% mill mud, red circle as 3 biological replicates) is separated from mill mud before soil application (purple) and soil samples without mill mud (blue and green). Based on the heat map (Fig 4B), the factory mill mud (before greenhouse experiments) was dominated by Firmicutes phylum (Clostridiaceae to Leuconostocaceae in uppermost rows) that survived the elevated temperatures (90°C for 1–2 h) during the raw sugar production process, as described previously [10]. In addition, Fig 4B shows lower levels of Proteobacteria from the family Pseuomonadaceae (that includes known plant pathogens [36]) in mill mud and mill mud-amended soil (2% MM) than the fertilized or control soils. Although this observation is promising for the fertilizer application of mill mud, strain-level phylogenetic resolution is necessary to distinguish pathogenic and nonpathogenic strains [37].

The results above suggest that mill mud was not the source of rhizobia, but contributed to the accumulation and resilience of rhizobia through the end of soybean growth cycle. Rhizobia could be triggered by the uptake of organic carbon in mill mud by symbionts [38] or through various other complex mechanisms involving signaling molecules [39], host specificity [40], nodule growth dynamics [41], host mutualism [42], and sanction [43]. Some members of the Firmicutes have been reported to enhance soybean growth [44], and mill mud-derived bacteria could affect plant growth directly or indirectly.

### Metagenomics phylogeny

**Fig 5:**
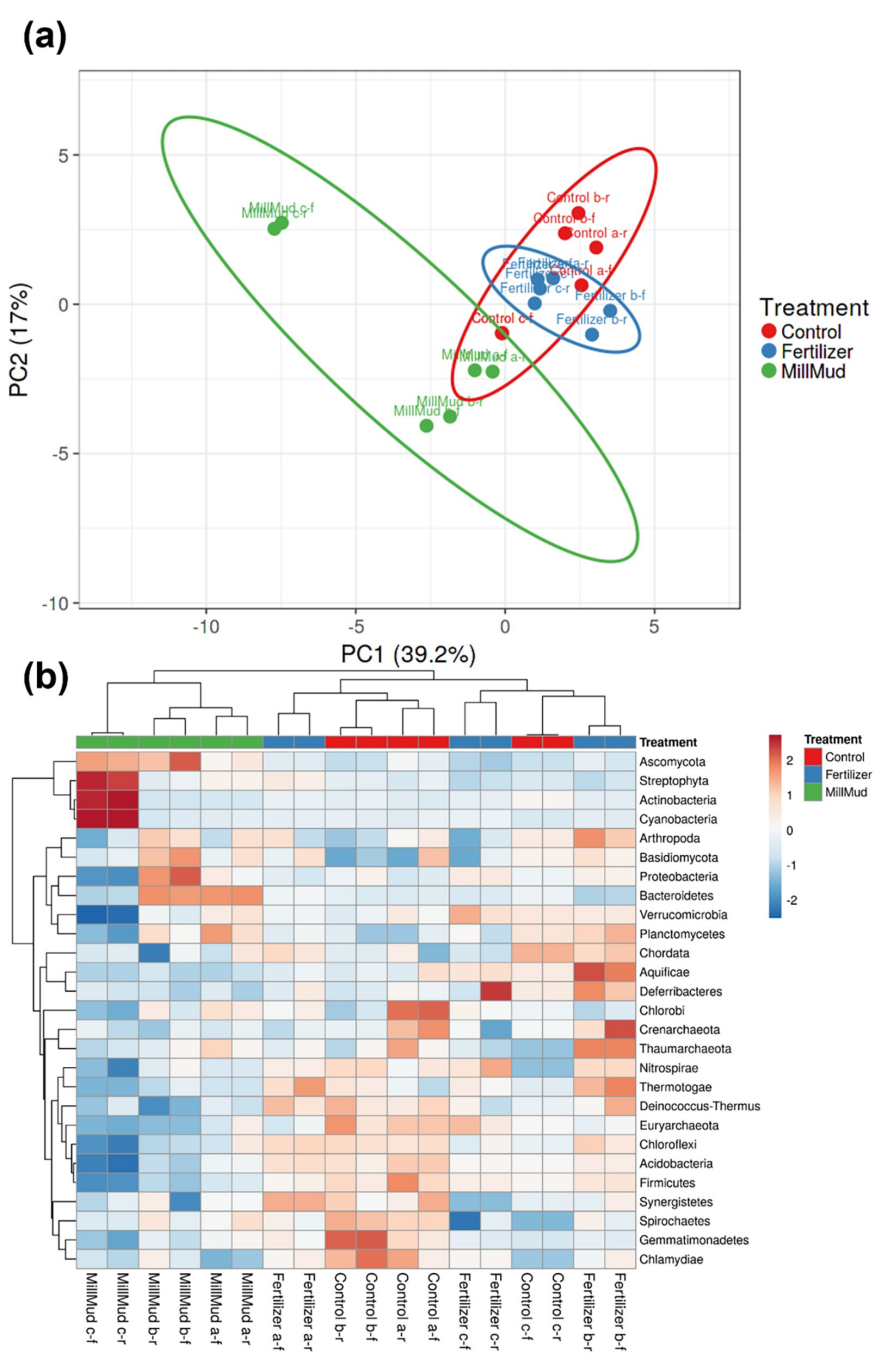
Phylogenetic affiliation of 27 phyla that accounted for more than 99% of metagenomic reads. The forward (columns labeled -f) and reverse (-r) reads were treated as separate technical replications for the 3 biological replicates.

Fig 5 includes a PCA (a) and heat map (b) based on the top 27 phylum level affiliations of more than 99% of the metagenomic reads. The control and the fertilizer treatment confidence intervals overlapped in the PCA, with the fertilizer samples clustering more tightly than the control. This suggests that fertilizer (blue points/eclipse in Fig 5A) exerted a strong selective pressure, driving the communities of these treatments in a similar direction. Decreased microbial diversity by the addition of chemical fertilizer (nitrate) has been reported [45]. The location of the mill mud amended samples in the PCA and the size of the confidence interval paints a less clear picture, owing in part, to within-treatment variation of the mill mud amended samples. According to the heat map, some of the drivers behind these differences (especially Millmud c in Fig 5B) are the presence of fungi (Ascomycota) and plant material (Steptophya) that were not detectable by 16S (Fig 4B). Also of interest were lower levels of nitrospirae and euryarcheota in the mill mud amended soils. Members of these taxa are known to oxidize ammonia to nitrate [46]. Ammonia (8% for 21% total nitrogen) and nitrate (13%) were the main sources of nitrogen in the chemical fertilizer, thus it is not surprising that organisms engaging in nitrification were enriched in the fertilizer treatment.

Although Firmicutes (clostridia, bacillus, and lactobacillus) dominated the fresh mill mud used in greenhouse experiments (3 far left columns in Fig 4B) both the 16S and the metagenomic phylogenetic analyses showed that relative abundance of Firmicutes were lower in mill mud amended soil than the control or fertilizer treatment (Firmicutes are shown in 5^th^ bottom row in Fig 5B). This suggests that the lasting effect that mill mud addition had on the soil microbiome was likely caused by either the nature of the mill mud itself (i.e. the organic matter and mineral composition), by some biological component of the microbiome added with mill mud, or by some tripartite interaction between the soybeans, the mill mud, and the microbial community.

### Metagenome function

**Fig 6:**
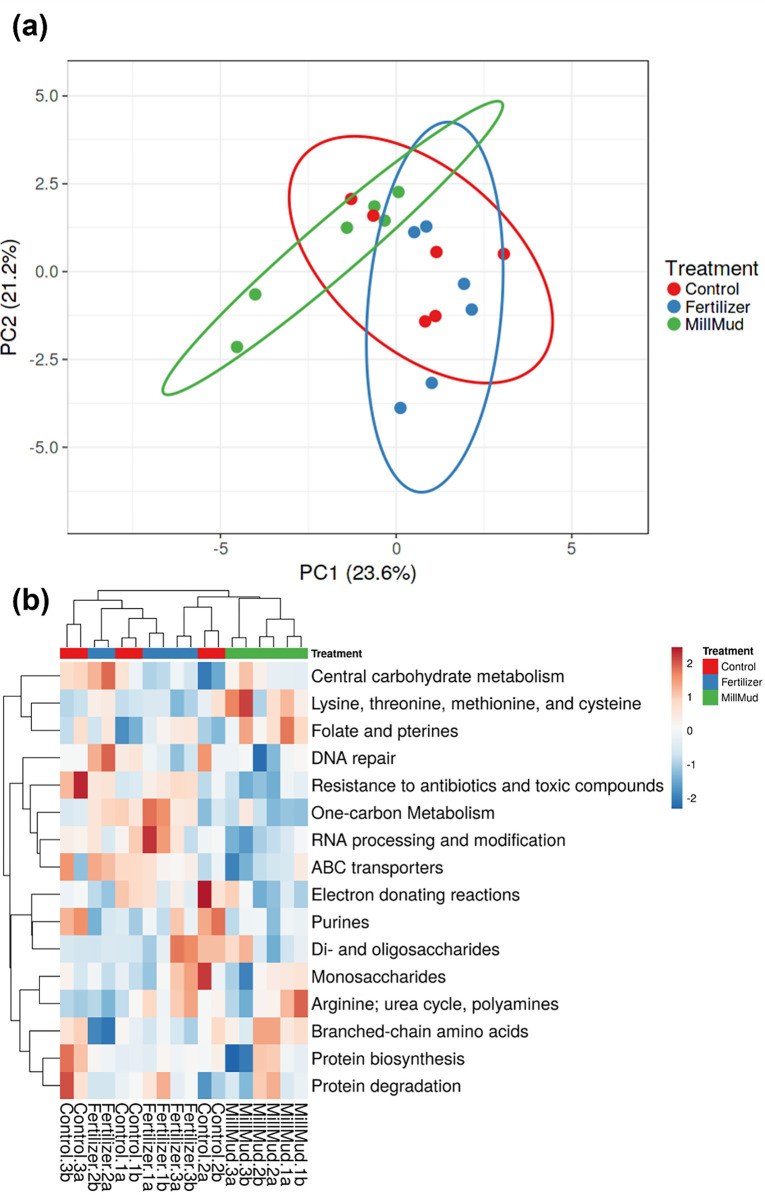
Metagenomic function.

Although all three treatments overlap to some extent in the PCA based on the 15 most abundant metagenome subsystems (Fig 6A), the mill mud treatment separated from fertilizer and control in the heatmap (Fig 6B). Of the dominant pathways in Fig 6B, RNA processing and modification was significantly lower in mill mud treatment than the control (after Bonferonni correction, p = 0.0023). Relative to the fertilizer treatment, genes related to resistance to antibiotics and toxic compounds (p = 0.00016) and methanogenesis (p = 0.0007) were lower, while genes encoding the synthesis of branched-chain amino acids (p = 0.0031) were higher in mill mud treatment (Fig 6B).

Antibiotic resistance in agricultural soils is a major One Health concern [47], as is the impact of agriculture on atmospheric methane. Recently, theoretical concerns have been raised about the impact of sugarcane vinasse application to soils [48] with respect to antibiotic resistance and methanogenesis [49]. Our microbiome-based results suggest that sugarcane mill mud appears to be preferable to traditional fertilizer with respect to these endpoints, though more direct analyses of actual antibiotic resistance and methane flux on a variety of soils receiving mill mud is needed to verify these results and determine how widely relevant they might be.

Taken together, our results demonstrate that mill mud was as effective as traditional fertilizing at increasing soybean pod yield in a Langford silt loam (Fig 1C). Mill mud treatment had the added benefit of increasing overall soil biodiversity (Fig 3), while reducing the biological potential for both antibiotic resistance and methanogenesis (Fig 6). Collectively, mill mud could induce host (soybean)-Rhizobia symbiosis in mineral soils, as evidenced by higher nodules by mill mud application than the chemical fertilizer (Fig 1B). Microbial consortium in applied mill mud could trigger resilience through complex mechanisms including cooperative functions and by enhancing the chance of soil/root colonization [5]. Systematic laboratory experiment is necessary to elucidate the mechanisms of symbiosis, while field trial could be used to test the influence of soil properties and other environmental factors.

Our results suggest a potential application of sugarcane mill mud as the feedstock material for potting mix, biostimulant, and related plant growth enhancers. This category of soil amendment, unlike chemical PKN fertilizer, offers benefits such as the plant stress resistance and nutrient use efficiency. There are several potential advantages of sugarcane mill mud as the plant growth stimulant over traditional feedstocks, e.g., peat, seaweed extract, humic substances, microbial inoculants, and protein extracts [50]. Firstly, we previously reported on the high fertilizer value and pasteurized nature of sugarcane mill mud from Louisiana and Florida [10]. Secondly, the present study showed that sugar-processing byproducts still maintain a variety of functional capacities as biostimulant to improve crop yields, as observed by the dominant enzymatic functions. Those functional attributes are necessary to justify the cost of applying biofertilizers and biostimulants. Specialized finished products like biostimulants may require advanced data presented in this study to show the functional capacities of sugarcane mill mud over commercial composts. Finally, the efficacy of mill mud will depend on its stability in the environment, especially with respect to the redox transformation and photolysis. The stability of mill mud throughout the product cycle (from shelf life to environmental fate) is an important aspect yet to be explored towards commercialization.

## Supporting information

S1 Data (XLSX)
